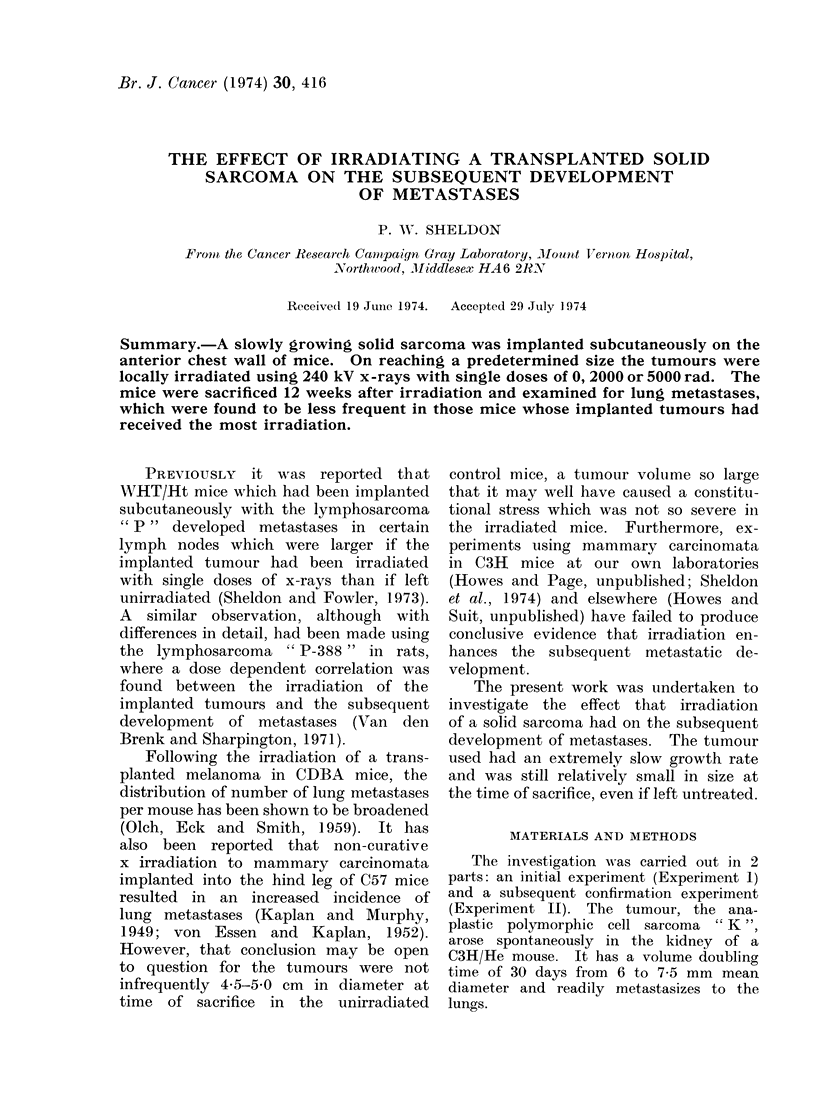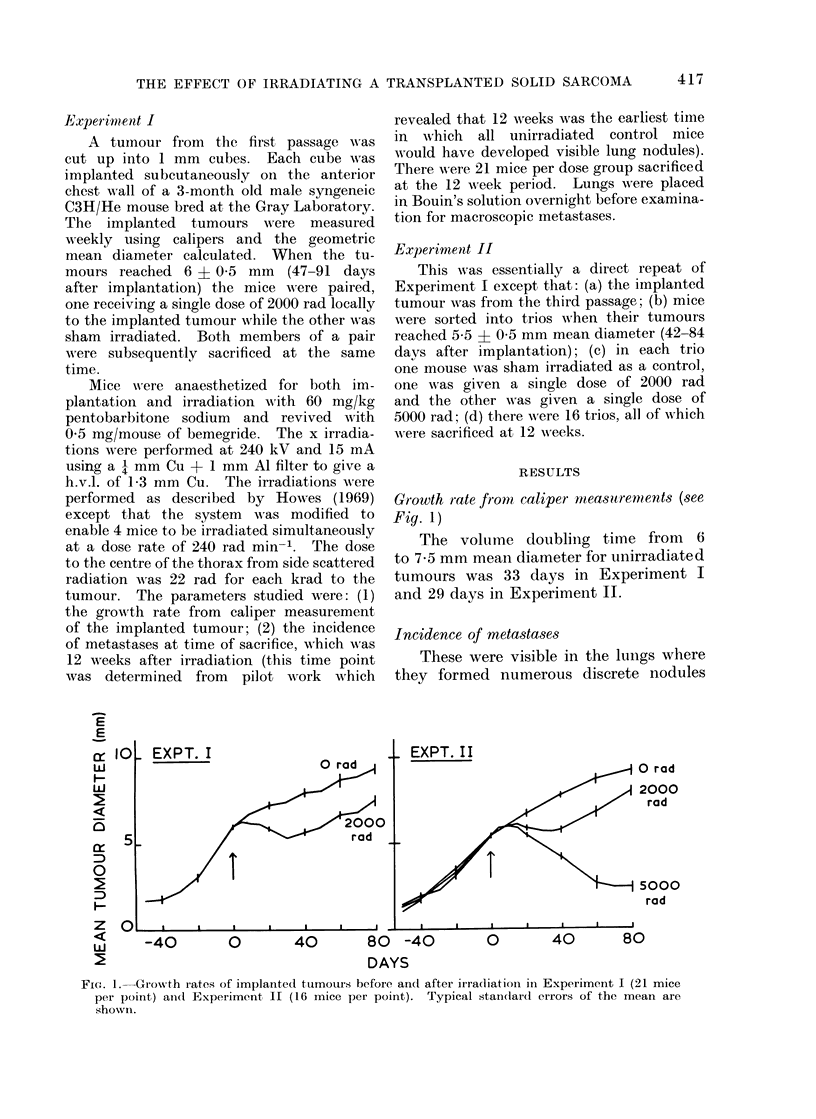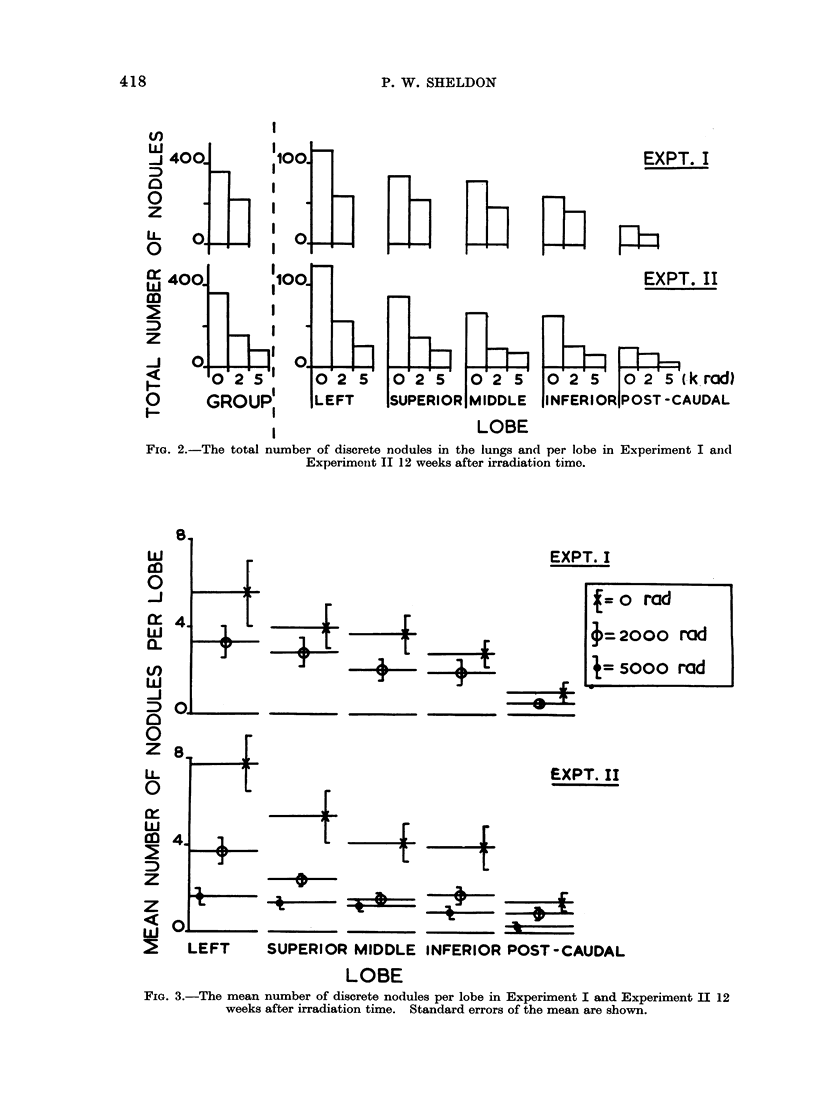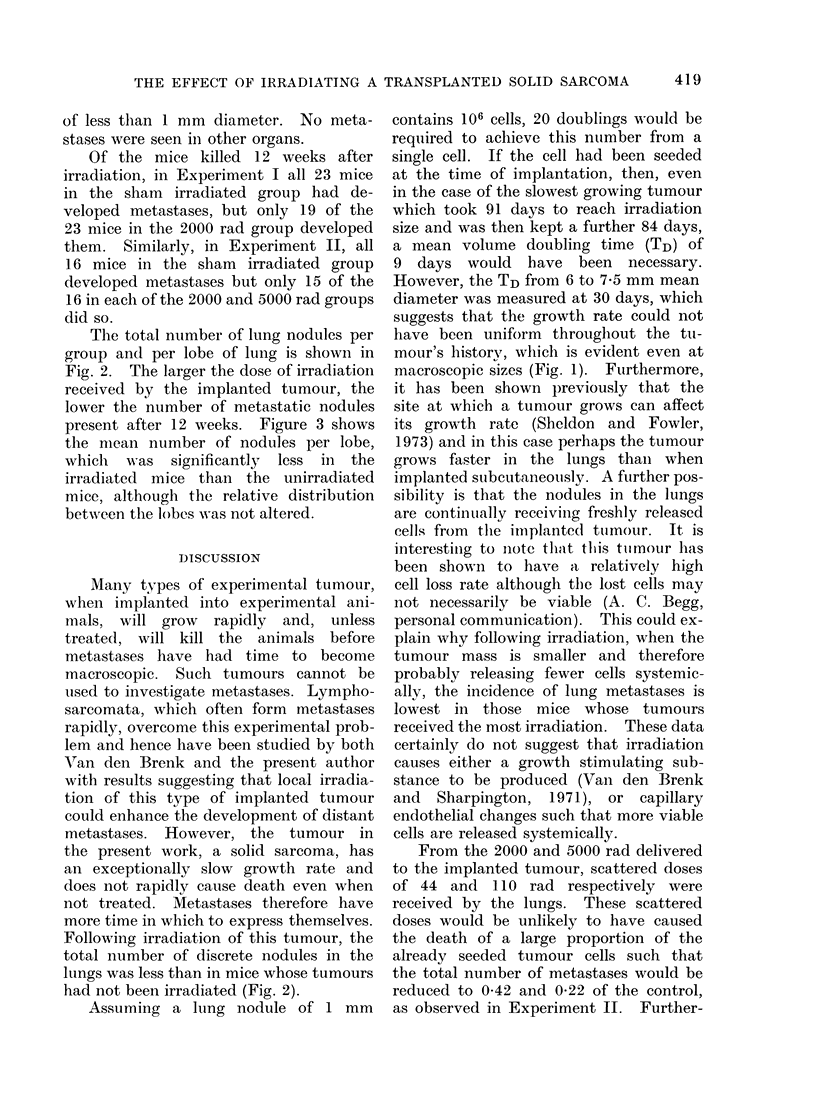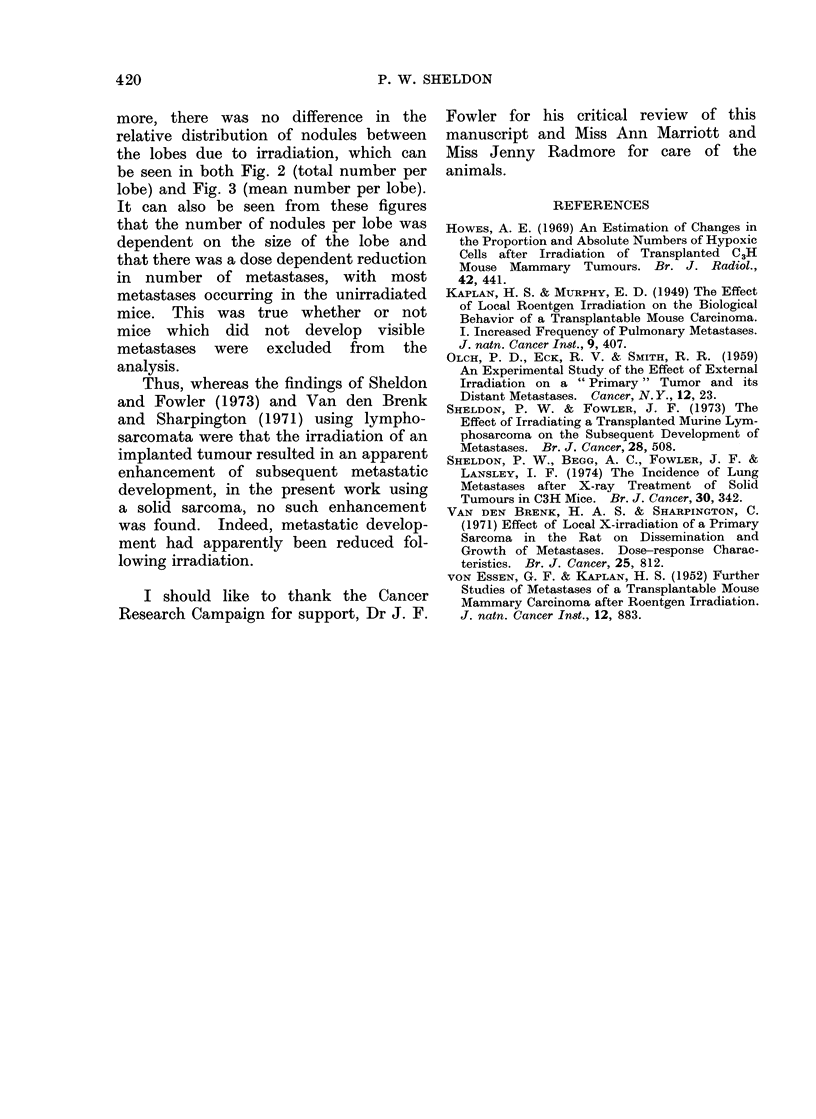# The Effect of Irradiating a Transplanted Solid Sarcoma on the Subsequent Development of Metastases

**DOI:** 10.1038/bjc.1974.215

**Published:** 1974-11

**Authors:** P. W. Sheldon

## Abstract

A slowly growing solid sarcoma was implanted subcutaneously on the anterior chest wall of mice. On reaching a predetermined size the tumours were locally irradiated using 240 kV x-rays with single doses of 0, 2000 or 5000 rad. The mice were sacrificed 12 weeks after irradiation and examined for lung metastases, which were found to be less frequent in those mice whose implanted tumours had received the most irradiation.


*Br. J. Cancer* (1974) **30**, 416

### THE EFFECT OF IRRADIATING A TRANSPLANTED SOLID SARCOMA ON THE SUBSEQUENT DEVELOPMENT OF METASTASES

P. W. SHELDON

*From the Cancer Research Campaign Gray Laboratory, Mount Vernon Hospital, Northwood, Middlesex HA6 2RN*

Received 19 June 1974. Accepted 29 July 1974

**Summary.—A slowly growing solid sarcoma was implanted subcutaneously on the anterior chest wall of mice. On reaching a predetermined size the tumours were locally irradiated using 240 kV x-rays with single doses of 0, 2000 or 5000 rad. The mice were sacrificed 12 weeks after irradiation and examined for lung metastases, which were found to be less frequent in those mice whose implanted tumours had received the most irradiation.**

PREVIOUSLY it was reported that WHT/Ht mice which had been implanted subcutaneously with the lymphosarcoma "P" developed metastases in certain lymph nodes which were larger if the implanted tumour had been irradiated with single doses of x-rays than if left unirradiated (Sheldon and Fowler, 1973). A similar observation, although with differences in detail, had been made using the lymphosarcoma "P-388" in rats, where a dose dependent correlation was found between the irradiation of the implanted tumours and the subsequent development of metastases (Van den Brenk and Sharpington, 1971).

Following the irradiation of a transplanted melanoma in CDBA mice, the distribution of number of lung metastases per mouse has been shown to be broadened (Olch, Eck and Smith, 1959). It has also been reported that non-curative x irradiation to mammary carcinomata implanted into the hind leg of C57 mice resulted in an increased incidence of lung metastases (Kaplan and Murphy, 1949; von Essen and Kaplan, 1952). However, that conclusion may be open to question for the tumours were not infrequently 4·5–5·0 cm in diameter at time of sacrifice in the unirradiated control mice, a tumour volume so large that it may well have caused a constitutional stress which was not so severe in the irradiated mice. Furthermore, experiments using mammary carcinomata in C3H mice at our own laboratories (Howes and Page, unpublished; Sheldon *et al.*, 1974) and elsewhere (Howes and Suit, unpublished) have failed to produce conclusive evidence that irradiation enhances the subsequent metastatic development.

The present work was undertaken to investigate the effect that irradiation of a solid sarcoma had on the subsequent development of metastases. The tumour used had an extremely slow growth rate and was still relatively small in size at the time of sacrifice, even if left untreated.

### MATERIALS AND METHODS

The investigation was carried out in 2 parts: an initial experiment (Experiment I) and a subsequent confirmation experiment (Experiment II). The tumour, the anaplastic polymorphic cell sarcoma "K", arose spontaneously in the kidney of a C3H/He mouse. It has a volume doubling time of 30 days from 6 to 7·5 mm mean diameter and readily metastasizes to the lungs.

*Experiment I*

A tumour from the first passage was cut up into 1 mm cubes. Each cube was implanted subcutaneously on the anterior chest wall of a 3-month old male syngeneic C3H/He mouse bred at the Gray Laboratory. The implanted tumours were measured weekly using calipers and the geometric mean diameter calculated. When the tumours reached $6 \pm 0{\cdot}5$ mm (47–91 days after implantation) the mice were paired, one receiving a single dose of 2000 rad locally to the implanted tumour while the other was sham irradiated. Both members of a pair were subsequently sacrificed at the same time.

Mice were anaesthetized for both implantation and irradiation with 60 mg/kg pentobarbitone sodium and revived with 0·5 mg/mouse of bemegride. The x irradiations were performed at 240 kV and 15 mA using a $\frac{1}{4}$ mm Cu + 1 mm Al filter to give a h.v.l. of 1·3 mm Cu. The irradiations were performed as described by Howes (1969) except that the system was modified to enable 4 mice to be irradiated simultaneously at a dose rate of 240 rad $min^{-1}$. The dose to the centre of the thorax from side scattered radiation was 22 rad for each krad to the tumour. The parameters studied were: (1) the growth rate from caliper measurement of the implanted tumour; (2) the incidence of metastases at time of sacrifice, which was 12 weeks after irradiation (this time point was determined from pilot work which revealed that 12 weeks was the earliest time in which all unirradiated control mice would have developed visible lung nodules). There were 21 mice per dose group sacrificed at the 12 week period. Lungs were placed in Bouin's solution overnight before examination for macroscopic metastases.

*Experiment II*

This was essentially a direct repeat of Experiment I except that: (a) the implanted tumour was from the third passage; (b) mice were sorted into trios when their tumours reached $5{\cdot}5 \pm 0{\cdot}5$ mm mean diameter (42–84 days after implantation); (c) in each trio one mouse was sham irradiated as a control, one was given a single dose of 2000 rad and the other was given a single dose of 5000 rad; (d) there were 16 trios, all of which were sacrificed at 12 weeks.

### RESULTS

*Growth rate from caliper measurements* (*see Fig.* 1)

The volume doubling time from 6 to 7·5 mm mean diameter for unirradiated tumours was 33 days in Experiment I and 29 days in Experiment II.

*Incidence of metastases*

These were visible in the lungs where they formed numerous discrete nodules

FIG. 1.—Growth rates of implanted tumours before and after irradiation in Experiment I (21 mice per point) and Experiment II (16 mice per point). Typical standard errors of the mean are shown.

FIG. 2.—The total number of discrete nodules in the lungs and per lobe in Experiment I and Experiment II 12 weeks after irradiation time.

FIG. 3.—The mean number of discrete nodules per lobe in Experiment I and Experiment II 12 weeks after irradiation time. Standard errors of the mean are shown.

of less than 1 mm diameter. No metastases were seen in other organs.

Of the mice killed 12 weeks after irradiation, in Experiment I all 23 mice in the sham irradiated group had developed metastases, but only 19 of the 23 mice in the 2000 rad group developed them. Similarly, in Experiment II, all 16 mice in the sham irradiated group developed metastases but only 15 of the 16 in each of the 2000 and 5000 rad groups did so.

The total number of lung nodules per group and per lobe of lung is shown in Fig. 2. The larger the dose of irradiation received by the implanted tumour, the lower the number of metastatic nodules present after 12 weeks. Figure 3 shows the mean number of nodules per lobe, which was significantly less in the irradiated mice than the unirradiated mice, although the relative distribution between the lobes was not altered.

### DISCUSSION

Many types of experimental tumour, when implanted into experimental animals, will grow rapidly and, unless treated, will kill the animals before metastases have had time to become macroscopic. Such tumours cannot be used to investigate metastases. Lymphosarcomata, which often form metastases rapidly, overcome this experimental problem and hence have been studied by both Van den Brenk and the present author with results suggesting that local irradiation of this type of implanted tumour could enhance the development of distant metastases. However, the tumour in the present work, a solid sarcoma, has an exceptionally slow growth rate and does not rapidly cause death even when not treated. Metastases therefore have more time in which to express themselves. Following irradiation of this tumour, the total number of discrete nodules in the lungs was less than in mice whose tumours had not been irradiated (Fig. 2).

Assuming a lung nodule of 1 mm contains $10^6$ cells, 20 doublings would be required to achieve this number from a single cell. If the cell had been seeded at the time of implantation, then, even in the case of the slowest growing tumour which took 91 days to reach irradiation size and was then kept a further 84 days, a mean volume doubling time ($T_D$) of 9 days would have been necessary. However, the $T_D$ from 6 to 7·5 mm mean diameter was measured at 30 days, which suggests that the growth rate could not have been uniform throughout the tumour's history, which is evident even at macroscopic sizes (Fig. 1). Furthermore, it has been shown previously that the site at which a tumour grows can affect its growth rate (Sheldon and Fowler, 1973) and in this case perhaps the tumour grows faster in the lungs than when implanted subcutaneously. A further possibility is that the nodules in the lungs are continually receiving freshly released cells from the implanted tumour. It is interesting to note that this tumour has been shown to have a relatively high cell loss rate although the lost cells may not necessarily be viable (A. C. Begg, personal communication). This could explain why following irradiation, when the tumour mass is smaller and therefore probably releasing fewer cells systemically, the incidence of lung metastases is lowest in those mice whose tumours received the most irradiation. These data certainly do not suggest that irradiation causes either a growth stimulating substance to be produced (Van den Brenk and Sharpington, 1971), or capillary endothelial changes such that more viable cells are released systemically.

From the 2000 and 5000 rad delivered to the implanted tumour, scattered doses of 44 and 110 rad respectively were received by the lungs. These scattered doses would be unlikely to have caused the death of a large proportion of the already seeded tumour cells such that the total number of metastases would be reduced to 0·42 and 0·22 of the control, as observed in Experiment II. Furthermore, there was no difference in the relative distribution of nodules between the lobes due to irradiation, which can be seen in both Fig. 2 (total number per lobe) and Fig. 3 (mean number per lobe). It can also be seen from these figures that the number of nodules per lobe was dependent on the size of the lobe and that there was a dose dependent reduction in number of metastases, with most metastases occurring in the unirradiated mice. This was true whether or not mice which did not develop visible metastases were excluded from the analysis.

Thus, whereas the findings of Sheldon and Fowler (1973) and Van den Brenk and Sharpington (1971) using lymphosarcomata were that the irradiation of an implanted tumour resulted in an apparent enhancement of subsequent metastatic development, in the present work using a solid sarcoma, no such enhancement was found. Indeed, metastatic development had apparently been reduced following irradiation.

I should like to thank the Cancer Research Campaign for support, Dr J. F. Fowler for his critical review of this manuscript and Miss Ann Marriott and Miss Jenny Radmore for care of the animals.

### REFERENCES

HOWES, A. E. (1969) An Estimation of Changes in the Proportion and Absolute Numbers of Hypoxic Cells after Irradiation of Transplanted $C_3H$ Mouse Mammary Tumours. *Br. J. Radiol.*, **42**, 441.

KAPLAN, H. S. & MURPHY, E. D. (1949) The Effect of Local Roentgen Irradiation on the Biological Behavior of a Transplantable Mouse Carcinoma. I. Increased Frequency of Pulmonary Metastases. *J. natn. Cancer Inst.*, **9**, 407.

OLCH, P. D., ECK, R. V. & SMITH, R. R. (1959) An Experimental Study of the Effect of External Irradiation on a "Primary" Tumor and its Distant Metastases. *Cancer, N.Y.*, **12**, 23.

SHELDON, P. W. & FOWLER, J. F. (1973) The Effect of Irradiating a Transplanted Murine Lymphosarcoma on the Subsequent Development of Metastases. *Br. J. Cancer*, **28**, 508.

SHELDON, P. W., BEGG, A. C., FOWLER, J. F. & LANSLEY, I. F. (1974) The Incidence of Lung Metastases after X-ray Treatment of Solid Tumours in C3H Mice. *Br. J. Cancer*, **30**, 342.

VAN DEN BRENK, H. A. S. & SHARPINGTON, C. (1971) Effect of Local X-irradiation of a Primary Sarcoma in the Rat on Dissemination and Growth of Metastases. Dose–response Characteristics. *Br. J. Cancer*, **25**, 812.

VON ESSEN, G. F. & KAPLAN, H. S. (1952) Further Studies of Metastases of a Transplantable Mouse Mammary Carcinoma after Roentgen Irradiation. *J. natn. Cancer Inst.*, **12**, 883.